# Identification of distinct circulating microRNAs in acute ischemic stroke patients with type 2 diabetes mellitus

**DOI:** 10.3389/fcvm.2022.1024790

**Published:** 2022-10-06

**Authors:** Salman M. Toor, Eman K. Aldous, Aijaz Parray, Naveed Akhtar, Yasser Al-Sarraj, Essam M. Abdelalim, Abdelilah Arredouani, Omar El-Agnaf, Paul J. Thornalley, Sajitha V. Pananchikkal, Ghulam Jeelani Pir, Raheem Ayadathil Thazhhe Kuni, Ashfaq Shuaib, Nehad M. Alajez, Omar M. E. Albagha

**Affiliations:** ^1^College of Health and Life Sciences (CHLS), Hamad Bin Khalifa University (HBKU), Qatar Foundation (QF), Doha, Qatar; ^2^Diabetes Research Center, Qatar Biomedical Research Institute (QBRI), Hamad Bin Khalifa University (HBKU), Qatar Foundation (QF), Doha, Qatar; ^3^The Neuroscience Institute, Academic Health System, Hamad Medical Corporation (HMC), Doha, Qatar; ^4^Qatar Genome Program, Qatar Foundation Research, Development and Innovation, Qatar Foundation (QF), Doha, Qatar; ^5^Neurological Disorders Research Center, Qatar Biomedical Research Institute (QBRI), Hamad Bin Khalifa University (HBKU), Qatar Foundation (QF), Doha, Qatar; ^6^Division of Neurology, Department of Medicine, University of Alberta, Edmonton, AB, Canada; ^7^Department of Neurology, Hamad Medical Corporation (HMC), Doha, Qatar; ^8^Translational Cancer and Immunity Center, Qatar Biomedical Research Institute (QBRI), Hamad Bin Khalifa University (HBKU), Qatar Foundation (QF), Doha, Qatar; ^9^Rheumatology and Bone Disease Unit, Centre for Genomic and Experimental Medicine, Institute of Genetics and Cancer, University of Edinburgh, Edinburgh, United Kingdom

**Keywords:** microRNA, miRNA, ischemic, stroke, diabetes mellitus, T2DM

## Abstract

Stroke is the second leading cause of global mortality and continued efforts aim to identify predictive, diagnostic, or prognostic biomarkers to reduce the disease burden. Circulating microRNAs (miRNAs) have emerged as potential biomarkers in stroke. We performed comprehensive circulating miRNA profiling of ischemic stroke patients with or without type 2 diabetes mellitus (T2DM), an important risk factor associated with worse clinical outcomes in stroke. Serum samples were collected within 24 h of acute stroke diagnosis and circulating miRNAs profiled using RNA-Seq were compared between stroke patients with T2DM (SWDM; *n* = 92) and those without T2DM (SWoDM; *n* = 98). Our analysis workflow involved random allocation of study cohorts into discovery (*n* = 96) and validation (*n* = 94) datasets. Five miRNAs were found to be differentially regulated in SWDM compared to SWoDM patients. Hsa-miR-361-3p and -664a-5p were downregulated, whereas miR-423-3p, -140-5p, and -17-3p were upregulated. We also explored the gene targets of these miRNAs and investigated the downstream pathways associated with them to decipher the potential pathways impacted in stroke with diabetes as comorbidity. Overall, our novel findings provide important insights into the differentially regulated miRNAs, their associated pathways and potential utilization for clinical benefits in ischemic stroke patients with diabetes.

## Introduction

Based on data gathered from 204 countries and territories between 1990 and 2019 (1), stroke remains the second leading cause of global mortality and has shown staggering increases in overall incidence, prevalence and death. These surges have been predominantly observed in younger populations belonging to low-income countries. At the same time, elevated blood pressure (BP), high body mass index (BMI), elevated fasting glucose levels, ambient particulate matter and smoking are considered the leading risk factors for stroke (1). In contrast, the incidence of ischemic strokes has been shown to steadily decline between 1993 and 2015 in studies conducted in the USA, with evidence of increased risk among Black and Hispanic females (≥70 years) in follow-up studies till 2019, while adults with treated BP also showed a significant reduction (42%) in stroke risk (2).

Stroke refers to a neurological deficit caused by acute focal injury of the central nervous system (CNS) due to a vascular cause (3). It is broadly classified into ischemic and hemorrhagic strokes, with the former caused by arterial occlusions, constituting most of stroke cases (4). Ischemic strokes are further divided into small-vessel occlusions, large-artery atherosclerosis, cardioembolism, or strokes of other determined or undermined etiologies (5). Treatment options for ischemic strokes are dependent on the duration from stroke onset, the extent of neurologic deficit and observations recorded by neuroimaging (3), while the clinical outcomes are widely assessed by the modified Rankin scale (mRS), which is also utilized as a robust tool for assessing the efficacy of treatment (6). Importantly, while diabetes is a known important risk factor for stroke (7), evidence has shown that it is also associated with worse clinical outcomes and fatal ischemic strokes (8, 9).

Diabetes is strongly associated with vascular diseases (10) and uncontrolled hyperglycemia can lead to ischemic or hemorrhagic strokes (11). In a 20-year follow-up data of more than 13,000 subjects, the risk of stroke was two- to threefold higher in type 2 diabetes mellitus (T2DM) patients, independently of other known risk factors (12). A meta-analysis covering ∼360,000 individuals showed that around one third of all stroke patients had diabetes and the presence of hyperglycemia/diabetes were associated with poor stroke outcomes (13). Notably, hyperglycemia included both pre-existing diabetes as comorbidity and post-stroke surges in fasting-blood glucose levels in stroke patients (13). Similarly, newly diagnosed diabetes cases were associated with more severe strokes, poorer outcomes, and increased mortality than pre-existing diabetes (14). In addition, the impact of pre-existing diabetes, acute hyperglycemic events during a stroke and post-reperfusion outcomes in stroke patients with diabetes can also have significant implications on stroke management (15). Fasting hyperglycemia recorded a day after mechanical thrombectomy in acute ischemic stroke patients was associated with worse clinical outcomes (16). Consequently, the associations between administration of anti-diabetic drugs and stroke risk have been thoroughly explored and evidence shows that selective anti-diabetic therapies such as metformin can have favorable effects on reducing stroke risk, while others may increase or pose no effect on the risk of ischemic strokes (17, 18). Additionally, investigations on the effects of cholesterol-lowering statins for reducing stroke risk showed elevated risk for diabetes due to statin use. However, cardiovascular events and clinical outcomes were favorable and exceeded the risk-to-benefit ratio for diabetes in favor of cardiovascular and mortality benefits of statin therapy (19, 20).

A stroke results in molecular imbalances due to the pathological and physiological events that take place. MicroRNAs (miRNAs) are small non-coding RNAs that form a regulatory network by affecting gene expression and are altered in various pathological conditions (21). Performing next-generation sequencing (NGS) on peripheral blood samples is a robust approach to decipher the global miRNA expression profiles (miRnome) and is increasingly utilized to improve understanding of various clinical pathologies including aneurysmal subarachnoid hemorrhages (22) and chronic kidney disease (23). Notably, miRNAs are consistently being explored as predictive, diagnostic, prognostic, and therapeutic markers in stroke (24). Accumulating evidence has shown the differential gradients in the expression levels of various miRNAs incurred transiently during ischemic strokes or with long-term alterations in peripheral blood samples from stroke patients (25–34). Likewise, the miRNA regulatory network affected in diabetes and its complications has been extensively explored and has led to the identification of various candidate miRNAs with potential diagnostic and prognostic ability (35, 36). However, very few studies have explored and linked the aberrant expression of miRNAs in diabetes and stroke (37–40). Importantly, these studies have exclusively utilized targeted approaches using real-time quantitative PCR. Combined, the impact of diabetes on stroke and the associated fluctuations in the miRNA regulatory network warrant comprehensive investigations for their potential to further improve our understanding of the molecular pathways involved in diabetes-associated stroke.

In the present study, the circulating miRNAs in serum samples from acute ischemic stroke patients with or without clinically diagnosed T2DM were profiled using RNA-Seq and thoroughly investigated. We used a rigorous analysis approach for the robust identification of differentially expressed miRNAs. Using a similar approach, we have recently reported a panel of 10 differentially regulated miRNAs with remarkably high discriminatory performance between acute ischemic stroke patients and healthy controls (41). In the present study we focused our investigations on T2DM, a critical factor associated with stroke outcomes. We identified a panel of five differentially regulated miRNAs between stroke patients with T2DM (SWDM) and stroke patients without T2DM (SWoDM). We also probed the previously experimentally validated gene targets and potential pathways affected by these miRNAs. Our findings warrant further functional investigations and validations for their ultimate clinical translation.

## Materials and methods

### Patient samples

**FIGURE 1:**
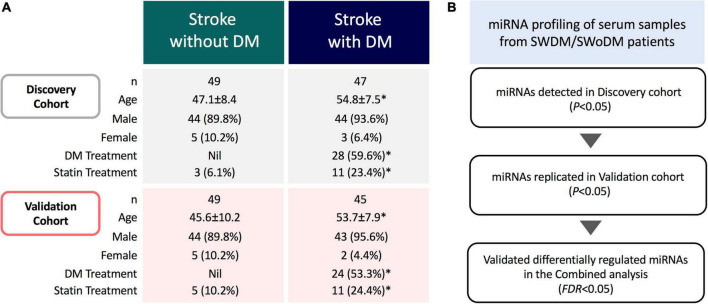
Study cohort and analysis workflow. **(A)** The study comprised acute ischemic stroke patients with and without type 2 diabetes mellitus (SWDM; *n* = 92, SWoDM; *n* = 98). Patients were randomly divided into discovery and validation cohorts with comparable distribution of covariates (age, gender, diabetes and statin treatment). Statistically significant parameters (*P* < 0.05) between SWDM and SWoDM patients are marked with an asterisk (*). **(B)** Serum samples were collected within 24 h of clinical diagnosis of acute ischemic stroke from SWDM and SWoDM patients. Differentially regulated and statistically significant miRNAs detected in the discovery dataset generated from miRNA transcripts by RNA-Seq were investigated in the validation dataset. Combined analysis was then performed to identify FDR-significant miRNAs presented as validated differentially regulated miRNAs in SWDM compared to SWoDM patients.

This study was approved by the Institutional Review Boards of Qatar Biomedical Research Institute (Approval no. 2019-013) and Hamad Medical Corporation (Approval no. 15304/15), Doha, Qatar. Written informed consents were taken from all participating individuals prior to sample collection. The study population (*n* = 190) comprised clinically diagnosed patients with acute ischemic stroke admitted to Hamad General Hospital (Doha, Qatar). Fresh serum samples were collected (within 24 h of stroke onset) and stored at −80°C for downstream analysis. Patients were divided into two groups based on clinical diagnosis of T2DM; SWDM (*n* = 92) and SWoDM (*n* = 98). Patients’ clinical records covering information on prior treatments administered for T2DM or cholesterol-lowering drugs (statins) were also retrieved from hospital records. The characteristic features of study population are presented in Figure 1A.

### Study design and analysis workflow

The overall study cohort comprised acute ischemic stroke patients with and without T2DM. The study cohort was randomly divided into discovery and validation cohorts with comparable distribution of covariates (number, gender, age, and treatment for T2DM and statin administration), and analyzed by RNA-Seq (*n* = 190) (Figure 1A). Briefly, the miRNA profiles of SWDM patients were first compared with SWoDM patients in the discovery cohort. The panel of differentially regulated and statistically significant (*P* < 0.05) miRNAs identified in the discovery dataset was validated in the validation cohort (Figure 1B). Combined analysis was then performed using more stringent analysis criteria (false discovery rate; FDR < 0.05) to identify robust differentially regulated miRNAs. This panel of miRNAs represented the differentially regulated miRNAs in SWDM compared to SWoDM and further downstream analyses were performed to explore the potential pathways affected by their targets.

### MicroRNA purification and sequencing

RNA-Seq was performed on collected samples as previously described (41). Briefly, circulating miRNA from serum samples (200 μl) were extracted using miRNeasy Serum/Plasma Advanced Kit (Qiagen, Hilden, Germany) and RNA concentrations were measured by Qubit RNA Broad Range Assay Kit (Invitrogen, CA, USA). Library preparation was carried out using QIAseq miRNA NGS Library Kit (Qiagen) and indexing was done using QIAseq miRNA NGS 96 Index IL kit (Qiagen). The quality control measures for generated libraries were performed using Qubit dsDNA HS assay kit (Invitrogen) and Agilent 2100 Bioanalyzer DNA1000 chip (Agilent Technologies, Santa Clara, CA, USA). The pooled libraries were clustered using TruSeq PE Cluster Kit v3-cBot-HS (illumina, San Diego, CA, USA). Sequencing was performed on illumina HiSeq 4000 system (10 million reads per sample) using HiSeq 3000/4000 SBS kit (illumina).

### Data processing

The NGS data generated as single reads (at 75 cycles) were aligned to the human miRbase v22 reference genome in CLC Genomics Workbench (v.21.0.5, Qiagen). The expression levels of miRNA transcripts were presented as counts per million (CPM) of the total count of mapped miRNA reads. Calibration for RNA spike-in (RNA transcript of known sequence and quantity) was also performed. The differential miRNA expression analyses were carried out on RStudio (version 4.1.1; RStudio, MA, USA) utilizing the DSEq2 method (V. 1.32.0) (42), while adjusting for covariates (age, gender, diabetes, and statin treatment). Statistical analyses and data visualization were performed using GraphPad Prism 9.1.2 (GraphPad Software, MD, USA).

### MicroRNA target and pathway analysis

The miRNA targets were identified from the miRTargetLink 2.0 (43). The gene enrichment and functional protein association network analysis of the target gene panel was performed by STRING (44), while functional pathway analyses were performed using QIAGEN Ingenuity Pathway Analysis (IPA) software (QIAGEN Inc.^1^) (45).

## Results

### Identification of differentially regulated microRNAs in stroke patients with type 2 diabetes mellitus

Our study cohort comprised clinically diagnosed stroke patients with or without T2DM as a comorbidity (Figure 1A). The study population was predominantly comprised of males, while SWDM patients were also significantly older than SWoDM patients. In addition, T2DM and statin therapies were administered to significantly higher proportions of SWDM compared to SWoDM patients, as expected. Considering these differences in characteristics of study cohorts, we corrected for these covariates in our analysis model and workflow, which involved random allocation of SWDM and SWoDM patients into discovery and validation datasets for the identification of replicated and statistically significant (FDR < 0.05) differentially regulated miRNAs (Figure 1B).

**FIGURE 2:**
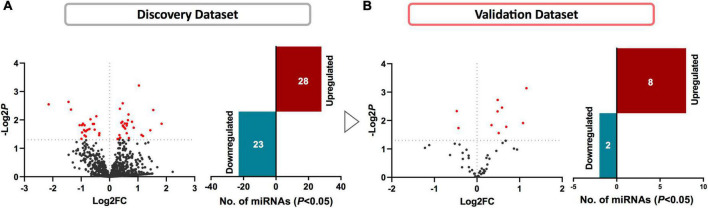
Identifying differentially regulated miRNAs in SWDM compared to SWoDM patients. Volcano plots show the number of differentially regulated miRNAs in SWDM versus SWoDM patients: red dots depict statistically significant (*P* < 0.05) and gray dots depict unsignificant miRNAs, while bar plots show the number of statistically significant miRNAs: downregulated in teal and upregulated presented in crimson color in **(A)** discovery and **(B)** validation datasets.

We first compared the circulating miRNA profiles of SWDM patients with SWoDM patients in the discovery cohort datasets (Figure 2A). We found that 51 miRNAs were differentially regulated between the two groups (*P* < 0.05) and showed varying degrees of fold change (FC) (Supplementary Table 1). We then tested these miRNAs in the validation cohort (Figure 2B) and out of the 51 miRNAs, 10 miRNAs showed significant dysregulation in the validation dataset (*P* < 0.05). Next, we performed combined analysis and five miRNAs remained statistically significant at FDR < 0.05 for the expression levels of the identified miRNA panel between SWDM and SWoDM patients with consistent direction of effect as shown in Table 1.

**TABLE 1:** Differentially regulated miRNAs in SWDM compared to SWoDM patients.

miRNA	Discovery		Validation		Combined	
	FC*	*P*-value	FC	*P*-value	FC	*P*-value**
hsa-miR-361-3p	−1.28	2.95 × 10^–2^	−1.39	4.67 × 10^–3^	−1.34	1.57 × 10^–4^
hsa-miR-423-3p	1.45	1.34 × 10^–2^	1.40	4.76 × 10^–3^	1.37	5.53 × 10^–4^
hsa-miR-664a-5p	−1.38	7.43 × 10^–3^	−1.35	1.85 × 10^–2^	−1.29	3.44 × 10^–3^
hsa-miR-140-5p	1.39	1.57 × 10^–2^	1.40	1.88 × 10^–3^	1.24	5.20 × 10^–3^
hsa-miR-17-3p	1.60	4.35 × 10^–2^	1.61	1.67 × 10^–2^	1.45	5.23 × 10^–3^

*Fold change. **FDR < 0.05.

### Differentially regulated microRNAs validated in stroke patients with type 2 diabetes mellitus

**FIGURE 3:**
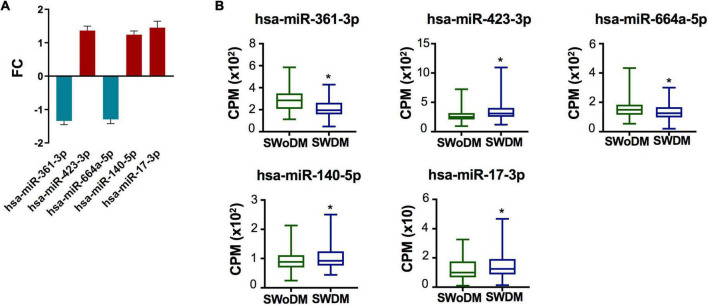
Validated miRNAs in SWDM patients compared to SWoDM patients. **(A)** Column plot shows the fold change (FC) and standard error of the mean (SEM) of FDR-significant validated, differentially regulated miRNAs (*n* = 5) in SWDM versus SWoDM patients: downregulated in teal and upregulated in crimson color. **(B)** Box and whiskers plots show the difference in counts per million (CPM) of the five validated miRNAs in SWDM and SWoDM patients. Mean with minimum and maximum values, upper and lower quartiles, and statistical significance (*P* < 0.05) marked by an asterisk (*) are shown for each dataset.

Five miRNAs; hsa-miR-361-3p, hsa-miR-423-3p, hsa-miR-664a-5p, hsa-miR-140-5p, and hsa-miR-17-3p were dysregulated between SWDM and SWoDM patients (Figure 3A). Out of these, two showed downregulation, while the remaining showed upregulation in SWDM versus SWoDM patients. Although these miRNAs showed moderate dysregulation in terms of FC, they showed high statistical significance (FDR < 0.05). Hsa-miR-361-3p was the most significant differentially regulated miRNA (Figure 3A). In addition, we also compared the CPM values of the five validated miRNAs, which also revealed significant differences between SWDM and SWoDM patients (Figure 3B).

### Identifying potential microRNA-mediated pathways affected in stroke patients with type 2 diabetes mellitus

**FIGURE 4:**
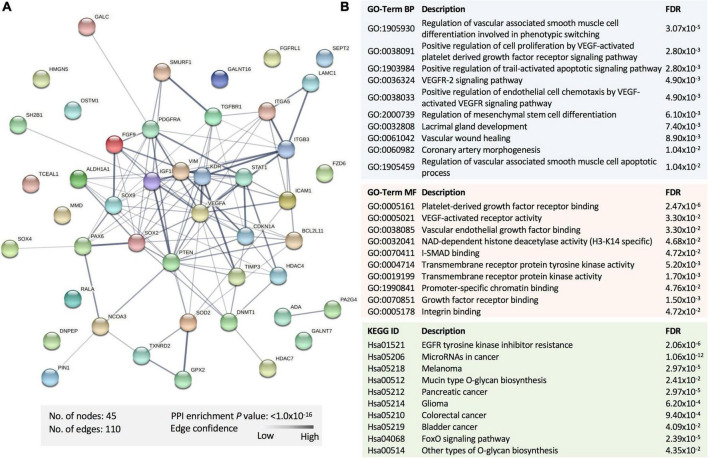
Functional enrichment analysis of the proteins encoded by the gene targets of dysregulated miRNAs in SWDM versus SWoDM patients. **(A)** The protein–protein interaction (PPI) network generated for the 47 gene targets of the identified miRNA panel is shown. Network nodes represent proteins, while edges depict protein–protein associations. The key network statistics are also presented. **(B)** The top functional enrichment annotations from Gene Ontology (GO), biological process (BP)/molecular function (MF), and Kyoto Encyclopedia of Genes and Genomes (KEGG) pathways are listed.

To explore the potential pathways affected by the panel of the statistically dysregulated five miRNAs in SWDM compared to SWoDM, we retrieved the experimentally validated molecular targets of these miRNAs in the miRTargetLink 2.0 database with strong experimental evidence. We compiled a list of 47 gene targets (Table 2). We first performed protein–protein interaction (PPI) and functional enrichment analysis of the proteins encoded by the 47 genes using STRING (Figure 4). The generated PPI network showed high statistical significance (*P* = 1.0 × 10^–16^) of the protein associations with strong involvement of VEGFA, STAT1, CDKN1A, and PTEN, among others (Figure 4A). Moreover, Gene Ontology (GO), biological process (BP), and molecular function (MF), and Kyoto Encyclopedia of Genes and Genomes (KEGG) pathway gene enrichment analysis predominantly showed vasculature-related pathways and the involvement of molecular pathways associated with disrupted homeostasis also observed in cancer (Figure 4B).

**TABLE 2:** The experimentally validated miRNA targets of the identified panel of differentially regulated miRNAs in SWDM compared to SWoDM patients.

miRNA	Target
hsa-miR-361-3p	*SH2B1*
hsa-miR-423-3p	*TCEAL1, CDKN1A, PA2G4, BCL2L11*
hsa-miR-664a-5p	*
hsa-miR-140-5p	*ALDH1A1, DNMT1, DNPEP, SOX2, HDAC4, VEGFA, PDGFRA, OSTM1, FGF9, TGFBR1, SOX9, FZD6, SEPT2, IGF1R, RALA, MMD, PAX6, HDAC7, LAMC1, ADA, SNORD12C, STAT1, PIN1, MEG3, GALC, GALNT16, SOX4, HMGN5, FGFRL1, SMURF1*
hsa-miR-17-3p	*ICAM1, KDR, VIM, SOD2, GPX2, TXNRD2, GALNT7, TIMP3, ITGA5, ITGB3, NCOA3, PTEN*

*No experimentally validated gene target.

**FIGURE 5:**
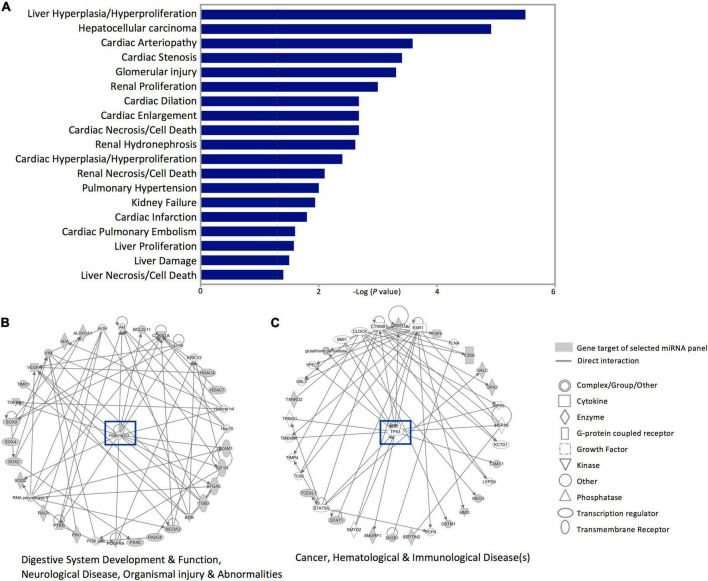
Disease annotation and Ingenuity pathway analysis for the gene targets of dysregulated miRNAs in SWDM versus SWoDM patients. The gene targets of the five dysregulated miRNAs in SWDM patients compared to SWoDM patients were analyzed for disease/function annotation and network analysis. **(A)** Bar plot shows the diseases annotations. **(B,C)** Ingenuity pathway network analysis of the gene targets of dysregulated miRNAs in SWDM patients are shown.

Next, investigating the clinical pathologies associated with these gene targets showed marked associations with cardiac anomaly-, hepatic-, and renal damage-related annotations (Figure 5A). In addition, the Ingenuity pathway and network analysis for the genes affected by dysregulated miRNAs in SWDM patients showed significant enrichment for two major pathways; histone H3 variants and *TP53* canonical pathways. Gene networks related to histone H3 highlighted enrichment in gastric development and function, neurological disease and organismal injury and abnormalities, which include edema, hemorrhage and lesions (Figure 5B). Additionally, cancer, hematological and immunological disease-related pathways, mediated by *TP53* gene network, were also annotated with interactions between miRNA panel gene targets (Figure 5C).

## Discussion

In this study, we identified five differentially regulated circulating miRNA in SWDM compared to SWoDM patients. While some of the miRNAs have been previously explored in relation to stroke or T2DM separately, their contribution to the impact of T2DM on stroke remains largely unexplored. SWDM patients have been previously reported to show worse disease outcomes, high recurrence and mortality compared to SWoDM patients (46). Deciphering the changes in the miRNA regulatory network in SWDM patients has, therefore, a potential therapeutic and prognostic significance.

Our data showed significant downregulation of miR-361-3p in SWDM compared to SWoDM. The downregulation of hsa-miR-361-3p has been previously reported in a cerebral artery occlusion-induced ischemic stroke murine model and presented as a potential therapeutic target following cerebral ischemic reperfusion injury (47). miR-361-3p has also been previously linked with vascular hemostasis. Upregulation of miR-361-3p was observed in patients with hereditary hemorrhagic telangiectasia (HHT) (48). Moreover, Huang et al., showed the involvement of miR-361-3p in inhibiting high-glucose induced vascular endothelial injury (49). Notably, the sole experimentally validated gene target of hsa-miR-361-3p, *SH2B1* is identified as a crucial protein involved in regulating energy balance, body weight, insulin sensitivity and glucose metabolism/homeostasis (50, 51). *SH2B1* is also associated with myocardial infarction in diabetic patients (52) while, Genome-Wide Association Studies (GWAS) have associated variants in *SH2B1* with BMI (53, 54). The dysregulation of miR-361-3p in SWDM patients indicates the impact on glucose metabolism but further studies are required to investigate this relation. Similarly, miR-664a-5p has been shown to promote neuronal differentiation (55) and its downregulation in SWDM patients indicates the suppression of neuroprotective machinery. Of note, dysregulation of miR-664a-5p has also been associated with the senescence of vascular smooth muscle cells and it has been proposed as a potential diagnostic marker and therapeutic target for cardiovascular diseases (56, 57). Moreover, Kim et al., reported upregulation of exosomal miR-664a-5p in obese T2DM patients compared to healthy controls (58). The dysregulation of miR-664a-5p could be associated with disease complications in SWDM patients but further investigations are required to confirm this association and also to experimentally validate its gene targets.

The downregulation of hsa-miR-423-3p has been previously reported as a biomarker for acute ischemic stroke patients (30) and has been associated with worse overall survival in patients with heart failure (59). However, its upregulation has been linked with onset and severity of Type 1 diabetes (60, 61) and it also showed high predictive potential in identifying T2DM remission after sleeve gastrectomy (62). We found that miR-423-3p was upregulated in SWDM patients compared to SWoDM patients, which suggests its association with diabetes. Importantly, upregulation of *CDKN1A*, an experimentally validated gene target of mir-423-3p, is presented as a specific marker of ischemic brain (63). GWAS also showed that variants in *CDKN1A* are associated with ischemic strokes (64), atrial fibrillation and cardioembolic stroke (65), while variants in other experimentally validated gene targets of mir-423-3p; *PA2G4* are associated with BMI (66) and *BCL2L11* with T2DM and cholesterol levels (67).

Ortega et al., reported upregulation of hsa-miR-140-5p in T2DM patients compared to healthy controls and showed its high discriminant capacity for T2DM (68). miR-140-5p was also upregulated in a blood stasis syndrome (BSS) model with diabetes compared to diabetes without BSS (69). Notably, it was reported that miR-140-5p could nullify the high glucose-induced inflammation and apoptosis in renal tubular cells (70) and mediate neuroprotection in ischemic strokes *via* exploitation of TLR4/NF-κB pathway (71). Of note, miR-140-5p has been also presented as an early biomarker for late-onset post-stroke depression (72). Our data showed upregulation of miR-140-5p in SWDM patients, which is in agreement with the findings of Ortega et al. (68) and suggests its involvement in diabetes-related pathways. Elevated activity of the experimentally validated gene target of mir-140-5p, *ALDH1A1* was associated with severity of T2DM (73). Among other experimentally validated miR-140-5p gene targets, *DNMT1*, *HDAC4*, and *HDAC7* are involved in epigenetic machinery. The associations between DNA methylation patterns and increased risk of various pathologies including diabetes, cancer, hypertension and atherosclerosis are well documented (74). The miRNA modulation of these gene targets coupled with modulation of additional gene targets associated with vasculature such as *VEGFA* and *MMD* by miR-140-5p indicates the potential involvement of another mechanism responsible for worse disease outcomes in SWDM patients *via* atherosclerotic vascular diseases or *via* epigenetic mechanisms (75). Additionally, GWAS have associated single nucleotide polymorphisms (SNPs) in miR-140-5p gene targets such as *SEPT2* (*SEPTIN2*) with T1DM (76), *DNMT1* with CVD (77) and *HDAC4* with increased susceptibility to myocardial infarction following coronary artery bypass surgery (78).

The upregulation of hsa-miR-17-3p was previously identified as a diagnostic and potential biomarker for acute ischemic strokes in two independent studies but it was not replicated in the validation cohorts (31, 79). Herein, we validated that miR-17-3p is upregulated in SWDM patients. miR-17-3p has been also associated with diabetic retinopathy (80, 81), coronary artery disease, cardiac ischemia (82–84) and has been previously presented as a circulating biomarker for T1DM (85, 86). Importantly, the experimentally validated gene target of miR-17-3p, *ICAM1* is strongly associated with poor prognosis in acute ischemic strokes (87). Changes in ICAM-1 serum concentration were reported in ischemic stroke patients with cerebral microbleeds and were associated with increased risk of hypertension and diabetes (88). Additionally, variants in *ICAM1* and serum ICAM-1 levels are also associated with the development of diabetes and diabetic nephropathy (89). However, targeting ICAM-1 in ischemic stroke patients is not a *via*ble therapeutic strategy as using anti-ICAM-1 antibody led to worse clinical outcomes in a clinical trial of 625 ischemic stroke patients (90). Among other gene targets of miR-17-3p, *VIM* has been associated with total/LDL-cholesterol measurement (91) and *PTEN* with T2DM (92) in GWAS. Combined, these data reflect the potential significance of miR-17-3p in ischemic strokes with T2DM and highlights pathways related to diabetic complications such as diabetic retinopathy.

To understand the potential clinical implication of the dysregulation in our miRNA panel in SWDM patients, we performed protein interaction network, functional gene enrichment and disease annotation and pathway analysis. The integration of functional annotations and disease mapping is widely followed and provides crucial understanding related to genes involved. miRNAs primarily regulate gene expression *via* repression during translation or degradation of target mRNA. The downregulation of miRNAs can promote expression of its target genes and consequently their protein-associated pathways. Conversely, miRNA upregulation can impede expression and function of target genes and their encoded proteins. However, since gene expression is influenced by various factors, functional validation of the effects of miRNAs on their targets is essential for confirmation.

Investigating the predicted interactions between proteins encoded by the gene targets of differentially regulated miRNAs in SWDM patients showed strong interaction enrichment. These interactions predominantly corresponded to vascular processes, mediated by *VEGFA*. Atherosclerosis is a known factor for impaired life expectancy, while diabetic nephropathy and retinopathy lead to renal diseases and blindness in diabetic patients (93). However, the disease complications observed in diabetic patients are multifaceted and involve modulation of multiple homeostasis-associated processes, also observed in various human malignancies. For instance, FoxO signaling regulates multiple processes such as cell cycle, apoptosis and metabolism, and is dysregulated in both cancer and diabetes (94). Of note, T2DM patients are at a higher risk of developing certain cancers including pancreatic and kidney cancer (95), potentially attributed to metabolic imbalances or genetic susceptibility. Our findings indicate the influence of diabetes-related complications and imbalances/exploitation of cancer-related pathways in SWDM patients.

Linking gene enrichment with disease and function annotations revealed associations with cardiac-, hepatic-, and renal-related pathologies, which are strongly associated with the clinical complications of diabetes. Physiological changes in cardiac and hepatorenal functions are also associated with stroke and imbalances in hematological indicators are commonly observed in diabetes and stroke patients. Moreover, the Ingenuity pathway analysis of the gene targets of dysregulated miRNAs in SWDM patients revealed gastric-, neurological-, and organismal injury-related pathways and cancer-, hematological-, and immunological disease-related pathways in SWDM patients. These networks encompass gastrointestinal disturbances, neurological deficits, inflammatory and immune imbalances in wound healing, atherosclerosis and vascular anomalies and reiterate the significance of underlying pathways, which are potentially exploited in SWDM patients and affect disease outcomes. Importantly, we identified several gene/protein targets which may be explored in future studies.

Investigating the delineation of the miRNA profiles of the ischemic brain from the healthy brain can potentially disclose critical pathways affected in stroke. However, the difficult accessibility to brain tissue renders investigating circulating miRNAs as the most feasible approach for use as disease biomarkers. Our findings provide insights into the differentially expressed miRNAs and their potential effects in SWDM compared to SWoDM patients. Importantly, our panel of differentially regulated miRNAs highlights the critical pathways potentially involved in the neuronal, cardiac, and diabetes-related complications observed in stroke patients with diabetes comorbidity and worse clinical outcomes. However, functional studies are warranted to investigate the biological significance of the identified dysregulated miRNAs and their associated pathways. Additionally, the relatively modest sample size of our study requires validation in a larger sample size in an external dataset. Of note, females represented a small proportion of our overall study cohort and repeating the analysis by excluding females generated essentially the same results. Overall, the gene targets of the panel of differentially expressed miRNAs and protein interactions uncovered in our study can be explored further for their clinical utilization for therapeutic benefits.

## Data availability statement

The datasets presented in this study can be found in online repositories. The names of the repository/repositories and accession number(s) can be found below: NCBI SRA, accession no. PRJNA879740 (https://www.ncbi.nlm.nih.gov/bioproject/PRJNA879740/).

## Ethics statement

The studies involving human participants were reviewed and approved by the Qatar Biomedical Research Institute and Hamad Medical Corporation, Doha, Qatar. The patients/participants provided their written informed consent to participate in this study.

## Author contributions

ST: formal analysis, visualization, and writing—original draft. EKA: data curation, formal analysis, and investigation. AP and NA: resources, investigation, and sample preparation. YA-S: investigation. EMA, AA, OE-A, and PT: resources. SP, GP, and RK: investigation and sample preparation. AS: resources, investigation, and writing—review and editing. NMA: formal analysis and writing—review and editing. OMEA: conceptualization, funding acquisition, project administration, resources, investigation, supervision, and writing—review and editing. All authors contributed to the article and approved the submitted version.

## Footnotes

1
https://digitalinsights.qiagen.com/IPA
